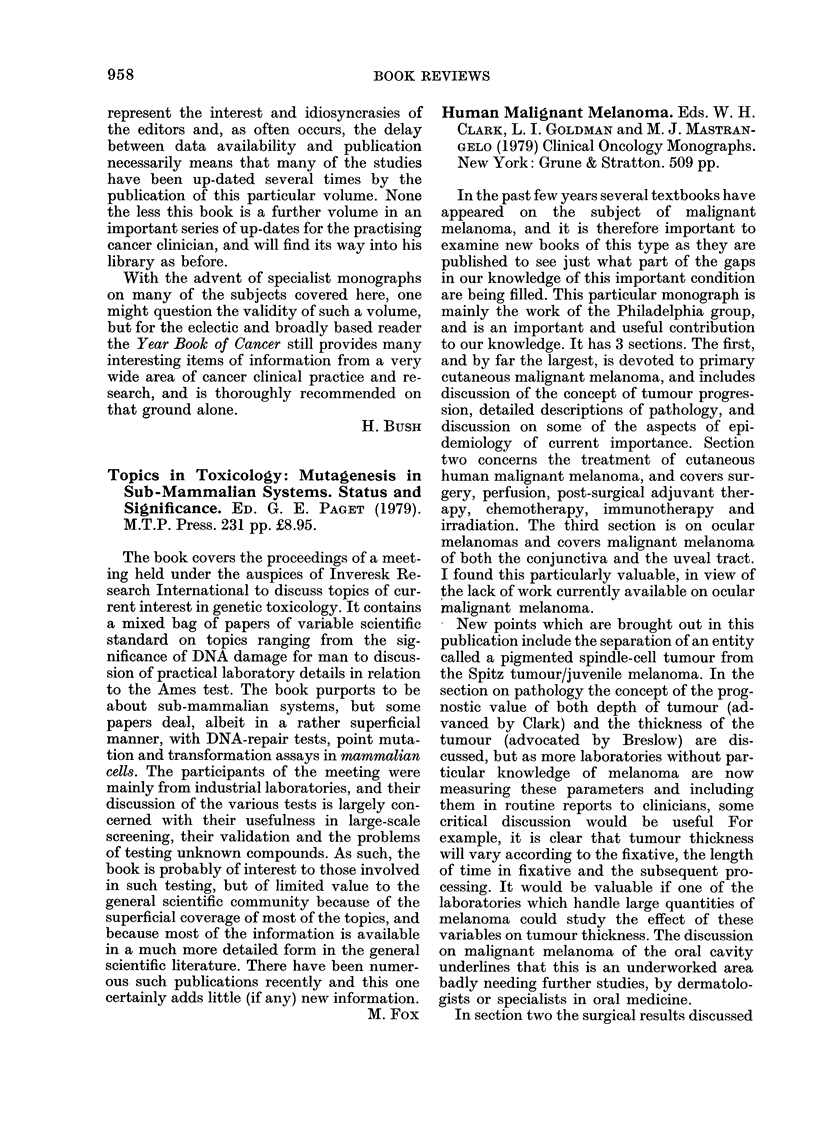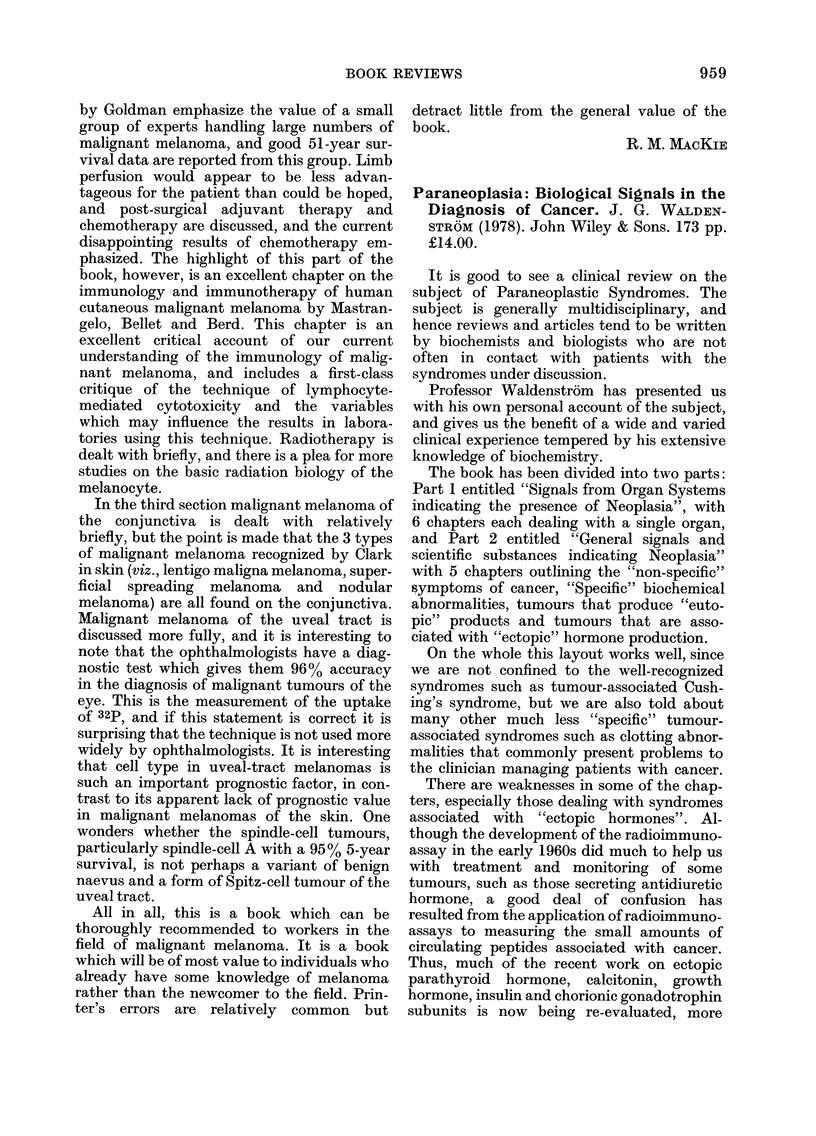# Human Malignant Melanoma

**Published:** 1979-12

**Authors:** R. M. MacKie


**Human Malignant Melanoma.** Eds. W. H. Clark, L. I. Goldman and M. J. Mastrangelo (1979) Clinical Oncology Monographs. New York: Grune & Stratton. 509 pp.

In the past few years several textbooks have appeared on the subject of malignant melanoma, and it is therefore important to examine new books of this type as they are published to see just what part of the gaps in our knowledge of this important condition are being filled. This particular monograph is mainly the work of the Philadelphia group, and is an important and useful contribution to our knowledge. It has 3 sections. The first, and by far the largest, is devoted to primary cutaneous malignant melanoma, and includes discussion of the concept of tumour progression, detailed descriptions of pathology, and discussion on some of the aspects of epidemiology of current importance. Section two concerns the treatment of cutaneous human malignant melanoma, and covers surgery, perfusion, post-surgical adjuvant therapy, chemotherapy, immunotherapy and irradiation. The third section is on ocular melanomas and covers malignant melanoma of both the conjunctiva and the uveal tract. I found this particularly valuable, in view of the lack of work currently available on ocular malignant melanoma.

New points which are brought out in this publication include the separation of an entity called a pigmented spindle-cell tumour from the Spitz tumour/juvenile melanoma. In the section on pathology the concept of the prognostic value of both depth of tumour (advanced by Clark) and the thickness of the tumour (advocated by Breslow) are discussed, but as more laboratories without particular knowledge of melanoma are now measuring these parameters and including them in routine reports to clinicians, some critical discussion would be useful For example, it is clear that tumour thickness will vary according to the fixative, the length of time in fixative and the subsequent processing. It would be valuable if one of the laboratories which handle large quantities of melanoma could study the effect of these variables on tumour thickness. The discussion on malignant melanoma of the oral cavity underlines that this is an underworked area badly needing further studies, by dermatologists or specialists in oral medicine.

In section two the surgical results discussed by Goldman emphasize the value of a small group of experts handling large numbers of malignant melanoma, and good 51-year survival data are reported from this group. Limb perfusion would appear to be less advantageous for the patient than could be hoped, and post-surgical adjuvant therapy and chemotherapy are discussed, and the current disappointing results of chemotherapy emphasized. The highlight of this part of the book, however, is an excellent chapter on the immunology and immunotherapy of human cutaneous malignant melanoma by Mastrangelo, Bellet and Berd. This chapter is an excellent critical account of our current understanding of the immunology of malignant melanoma, and includes a first-class critique of the technique of lymphocyte-mediated cytotoxicity and the variables which may influence the results in laboratories using this technique. Radiotherapy is dealt with briefly, and there is a plea for more studies on the basic radiation biology of the melanocyte.

In the third section malignant melanoma of the conjunctiva is dealt with relatively briefly, but the point is made that the 3 types of malignant melanoma recognized by Clark in skin (*viz.*, lentigo maligna melanoma, superficial spreading melanoma and nodular melanoma) are all found on the conjunctiva. Malignant melanoma of the uveal tract is discussed more fully, and it is interesting to note that the ophthalmologists have a diagnostic test which gives them 96% accuracy in the diagnosis of malignant tumours of the eye. This is the measurement of the uptake of $^{32}P$, and if this statement is correct it is surprising that the technique is not used more widely by ophthalmologists. It is interesting that cell type in uveal-tract melanomas is such an important prognostic factor, in contrast to its apparent lack of prognostic value in malignant melanomas of the skin. One wonders whether the spindle-cell tumours, particularly spindle-cell A with a 95% 5-year survival, is not perhaps a variant of benign naevus and a form of Spitz-cell tumour of the uveal tract.

All in all, this is a book which can be thoroughly recommended to workers in the field of malignant melanoma. It is a book which will be of most value to individuals who already have some knowledge of melanoma rather than the newcomer to the field. Printer's errors are relatively common but detract little from the general value of the book.

R. M. MacKie